# Ret finger protein-like 3 promotes tumor cell growth by activating telomerase reverse transcriptase expression in human lung cancer cells

**DOI:** 10.18632/oncotarget.2557

**Published:** 2014-11-13

**Authors:** Wangbing Chen, Jianjun Lu, Yu Qin, Jingshu Wang, Yun Tian, Dingbo Shi, Shusen Wang, Yao Xiao, Meng Dai, Lu Liu, Guo Wei, Taihua Wu, Bilian Jin, Xiangsheng Xiao, Tie-Bang Kang, Wenlin Huang, Wuguo Deng

**Affiliations:** ^1^ Sun Yat-sen University Cancer Center, State Key Laboratory of Oncology in South China, Collaborative Innovation Center of Cancer Medicine, Guangzhou, China; ^2^ Institute of Cancer Stem Cell & The First Affiliated Hospital, Dalian Medical University, Dalian, China; ^3^ Department of Thoracic Surgery, The First Affiliated Hospital, Sun Yat-sen University, Guangzhou, China; ^4^ Cancer Center, Union Hospital, Tongji Medical College, Huazhong University of Science and Technology, Wuhan, China; ^5^ State Key Laboratory of Targeted Drug for Tumors of Guangdong Province, Guangzhou Double Bioproduct Inc., Guangzhou, China

## Abstract

In this study, we identified ret finger protein-like 3 (RFPL3) as a hTERT promoter binding protein in lung cancer cells. The high hTERT promoter-binding activity of RFPL3 was detected in lung cancer cells compared to normal cells. Chromatin immunoprecipitation confirmed RFPL3 as a tumor-specific hTERT promoter binding protein. Overexpression of RFPL3 activated hTERT promoter and up-regulated hTERT expression and telomerase activity. Inhibition of RFPL3 expression by siRNA suppressed hTERT promoter activation and telomerase activity. Inhibition of RFPL3 by siRNA or shRNA also significantly inhibited tumor cell growth *in vitro* and in a xenograft mouse model *in vivo*. Immunohistochemical analysis of 181 human lung adenocarcinomas specimens showed a significant correlation between RFPL3 and hTERT expression. The overexpression of RFPL3 was also associated significantly with lymph node metastasis. Univariate and multivariate Cox model analyses of NSCLC clinical specimens revealed a strong correlation between RFPL3 expression and overall survival. These results demonstrate that RFPL3 is an important cellular factor which promotes lung cancer growth by activating hTERT expression and may be a potential novel therapeutic target for lung cancer.

## INTRODUCTION

Lung cancer is the leading cause of cancer-related death [1], and non–small cell lung cancer (NSCLC) accounts for approximately 80% of lung cancer diagnoses. Adenocarcinoma (AC) accounts for over 50% of all NSCLC and is the most frequently diagnosed subtype of NSCLC. Current therapeutic strategies for lung cancer include surgery, chemotherapy, radiotherapy and recently established molecular targeted therapies. Within the last decade, the discovery of targetable driver oncogenes such as EGFR mutations and ALK fusions have improved outcome for patients with NSCLC [2–5]. However, patients with these common mutations constitute only a small fraction of the entire population with advanced NSCLC. To date, the 5-year overall survival rate for patients with NSCLC has not been markedly improved [1, 6, 7]. Therefore, there is an urgent need for further understanding of the molecular mechanisms in lung cancer tumorigenesis and for identifying new therapeutic target to improve the prognosis of patients.

Human telomerase reverse transcriptase (hTERT) is over-expressed in 85–90% of cancers but is present at very low or almost undetectable levels in most normal cells [8]. Therefore, this cancer-specific property provides an ideal chance for us to utilize hTERT for cancer treatment. However, the current hTERT targeting therapeutics still showed minimal adverse effects on normal cells [8–10]. This may partially attribute to the fact that some normal cells, including male germline and some adult tissue stem cells, express relatively high levels of hTERT [11–13]. Recent studies suggest that the *hTERT* promoter activity itself appears to be relatively higher in hTERT-positive cancer cells than in hTERT-positive normal cells[14]. To exploit the cancer-specific activation mechanism of hTERT may develop new treatment strategies. Because the reactivation and upregulation of *hTERT* during carcinogenesis are primarily regulated at the transcriptional level [14], we speculated that the protein factors with cancer-specific *hTERT* promoter binding activity maybe become ideal therapeutic targets. In this study, we sought to discovery and identify the novel and tumor-specific *hTERT* promoter-regulating proteins in lung cancer cells.

The streptavidin-agarose pulldown assay is a useful and feasible approach for analyzing the binding of an array of proteins on DNA sequence [15–17]. The method combined with high-throughput proteomics can generate an effective screening system to indentify the novel DNA sequence binding proteins [18–21]. Using this technology, we previously discovered novel expression regulating mechanisms of carcinogenic genes *COX-2* [22–24]. In this study, we used this systematic approach to pull down the potential hTERT promoter-binding proteins in lung cancer cells and identified the one as ret finger protein-like 3 (RFPL3). *RFPL3* gene locates at human 22q12.3, and belongs to the RFPL proteins family which plays an important regulatory role in embryonic development [25, 26]. It has been reported that RFPL1 exerted its anti-proliferative activity through controlling cell-cycle progression in HeLa cells [27]. However, the expression and potential role of other RFPL family members including RFPL3 in tumors are unknown.

In the present study, we studied the role of RFPL3 in regulating *hTERT* promoter activity as well as hTERT expression. Both in *vitro* and in *vivo* functional assays were also conducted to characterize the biologic effects and potential molecular mechanisms of RFPL3 in lung tumorigenesis. The expression status and clinical significance of RFPL3 in lung adenocarcinomas was also investigated.

## RESULTS

### Pulldown and identification of RFPL3 as a *hTERT* promoter-binding protein

**Figure 1:**
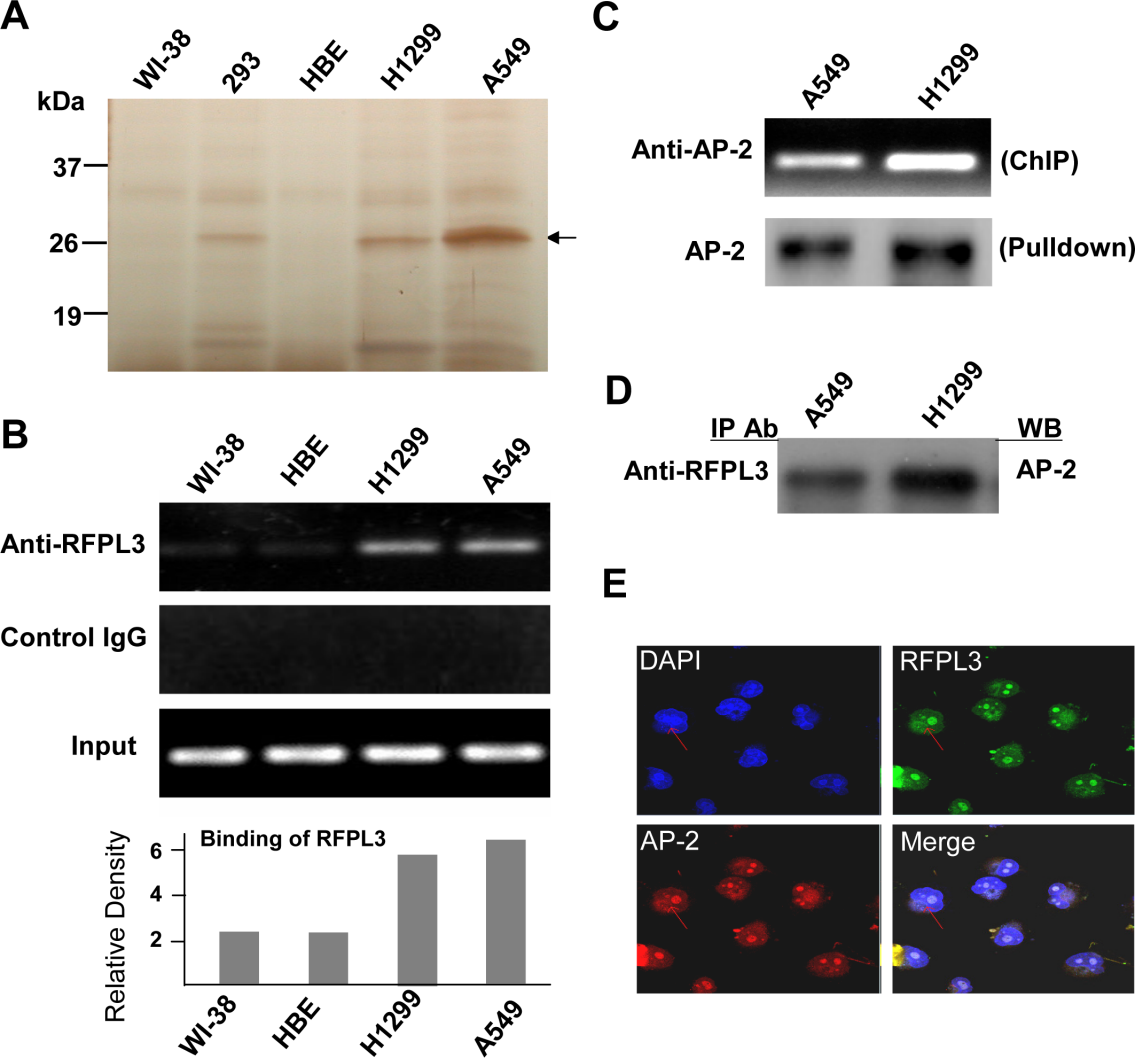
Pulldown and identification of tumor-specific *hTERT* promoter binding proteins **(A)** The potential *hTERT* promoter-binding proteins were pulled down, separated by the SDS-PAGE, and visualized by silver staining. A representative SDS-PAGE image is shown, and the arrow indicates the candidate *hTERT* promoter-binding protein. **(B)** Chromatin immunoprecipitation assays were carried out using the *hTERT* promoter from normal lung cells and lung cancer cells. PCR products of hTERT promoter (−378 to +60) were separated on 1% agarose gels. The IgG was used as a negative control. **(C)** Chromatin immunoprecipitation assays were done using antibody against AP-2. The PCR products of hTERT promoter (−378 to +60) were separated on 1% agarose gels. The streptavidin-agarose pulldown with hTERT promoter (−378 to +60) as probes was done. AP-2 was tested in the pulled down protein complex by immunoblot using antibody against AP-2. **(D)** The nuclear extracts of human lung normal and cancer cells were prepared for immunoprecipitation using an antibody against RFPL3 and then evaluated by immunoblot using antibody against AP-2. **(E)** Human lung cancer H1299 cells grown on chamber slides were cultivated for 24 h, and the subcellular localization and the colocalization of RFPL3 with AP-2 were examined by confocal microscopy analysis with a confocal microscope. Densitometric analysis was used to analyze quantitatively the binding of RFPL3 on hTERT promoter.

The streptavidin-agarose bead pulldown assay is a new approach to detect and discover the unknown promoter-regulating factors for the known target genes [23, 28]. In this study, we used this technology to pull down the novel and tumor-specific *hTERT* promoter regulating proteins in lung cancer cells. We synthesized a 5′-biotinylated 438-bp *hTERT* core promoter DNA probe and used lung cancer cells (H1299, A549) and normal lung cells (WI-38, HBE) as the models. After incubation of nuclear protein extracts with the *hTERT* promoter probe and streptavidin–agarose beads, the candidate complexes that specifically bound to the *hTERT* promoter probe were pulled down, separated by SDS-PAGE, and then visualized by silver staining. As shown in Fig. 1A (arrow), one of the protein bands (at approximately 27 kDa) was predominantly present in the lung cancer cells but almost undetectable in the normal lung cells. The protein bands of interests were dissected from the gel and identified by mass spectrum analysis. The candidate lung cancer-specific *hTERT* promoter-binding protein (Fig. 1A, arrow) was predicted to be ret finger protein-like 3 (RFPL3).

### Validation of RFPL3 as a *hTERT* promoter-binding protein

To verify that RFPL3 is a potential *hTERT* promoter-binding protein, the ChIP assay was used to detect the proteins on *hTERT* promoter. As shown in Fig. 1B, more RFPL3 proteins bound to the *hTERT* promoter in lung cancer cells (H1299, A549), but the binding was very weak in lung normal cells (WI-38, HBE).

We also determined whether RFPL3 functions in lung cancer cells by interacting with other transcription factors such as AP-2, an important protein which controls the expression of hTERT. The streptavidin-agarose pulldown and immunoblot analysis showed that AP-2 effectively bound to the hTERT promoter probe in lung cancer H1299 and A549 cells (Fig. 1C). The ChIP assay also confirmed the binding of AP-2 on the hTERT promoter structure (Fig. 1C). In addition, we also detected the interaction between RFPL3 and AP-2 proteins in lung cancer cells. Immunopreciptation analysis revealed that RFPL3 could interact directly with AP-2 in H1299 and A549 cells (Fig. 1D). The dual immunofluorescence analysis further demonstrated the co-localization of these two proteins (Fig. 1E). These results indicate that RFPL3 may regulate hTERT expression by interacting with the transcriptional factor AP-2 bound on the hTERT promoter in lung cancer cells.

### Upregulation of hTERT expression and telomerase activity by RFPL3 overexpression

**Figure 2:**
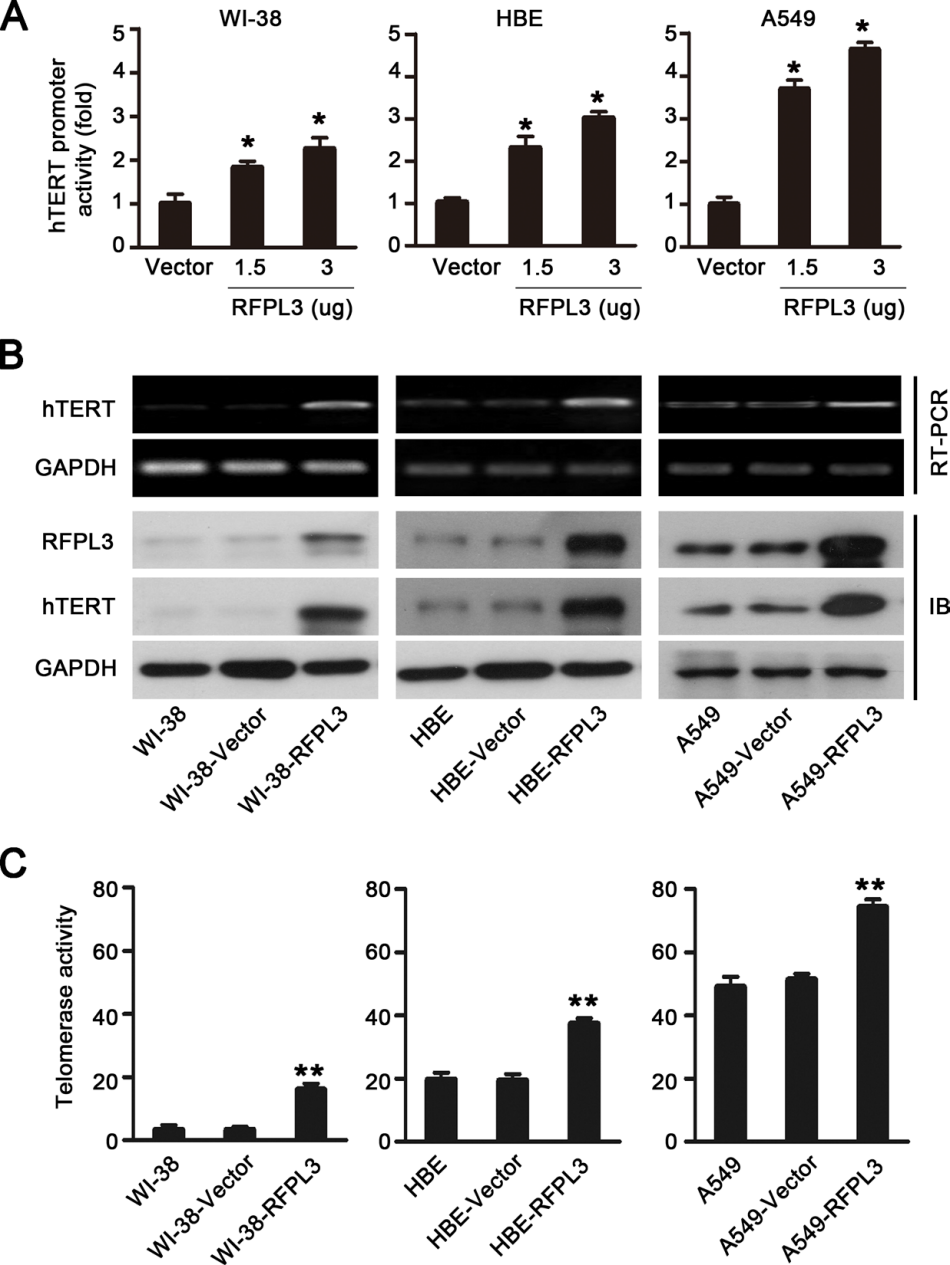
Overexpression of RFPL3 up-regulates *hTERT* promoter acivation and promotes hTERT expression and telomerase activity **(A)** WI-38, HBE and A549 cells were co-transfected with hTERT-luciferase and the indicated doses of pCDNA3.1 empty vector or pCDNA3.1-RFPL3 plasmid for 48 h. Luciferase activity was measured using a dual-luciferase assay. The activation of luciferase was calculated relative to cells transfected with empty vector. All of the measurements represent the means ± SE of three independent experiments (*P < 0.05). **(B)** WI-38, HBE and A549 cells were transfected with pCDNA3.1 empty vector or pCDNA3.1-RFPL3 plasmid for 48 h, and the expression of hTERT mRNA and protein was analyzed by RT-PCR and Western blot. **(C)**Telomerase activity was measured in WI-38, HBE and A549 cells using a telomeric repeat amplification protocol assay-based TeloTAGGG telomerase PCR enzyme-linked immunosorbent assay (**P < 0.01).

We next analyzed the effect of RFPL3 on *hTERT* promoter activity by dual-luciferase reporter assay. The normal lung cell lines (WI-38 and HBE) and lung cancer cell lines (A549) were co-transfected with the *hTERT* promoter driven-luciferase reporter and the plasmids expressing RFPL3. The results showed that *hTERT* promoter-driven luciferase activity was activated by RFPL3 in a dose-dependent manner (Fig. 2A).

Since the expression of the *hTERT* gene and the activation of telomerase are primarily regulated at the transcriptional level [14], we next confirmed the role of RFPL3 in regulating the transcription of hTERT by evaluating the effect of RFPL3 on endogenous hTERT mRNA and protein expression and telomerase activity in normal lung cell lines and lung cancer cell lines. We found that the overexpression of RFPL3 in normal lung cell lines (WI-38 and HBE) and lung cancer cell lines (A549) significantly increased the levels of hTERT mRNA and protein expression (Fig. 2B) and the activity of telomerase (Fig. 2C). These results suggest that RFPL3 overexpression up-regulates *hTERT* promoter activity and expression.

### Inhibition of hTERT expression and telomerase activity by RFPL3 knockdown

**Figure 3:**
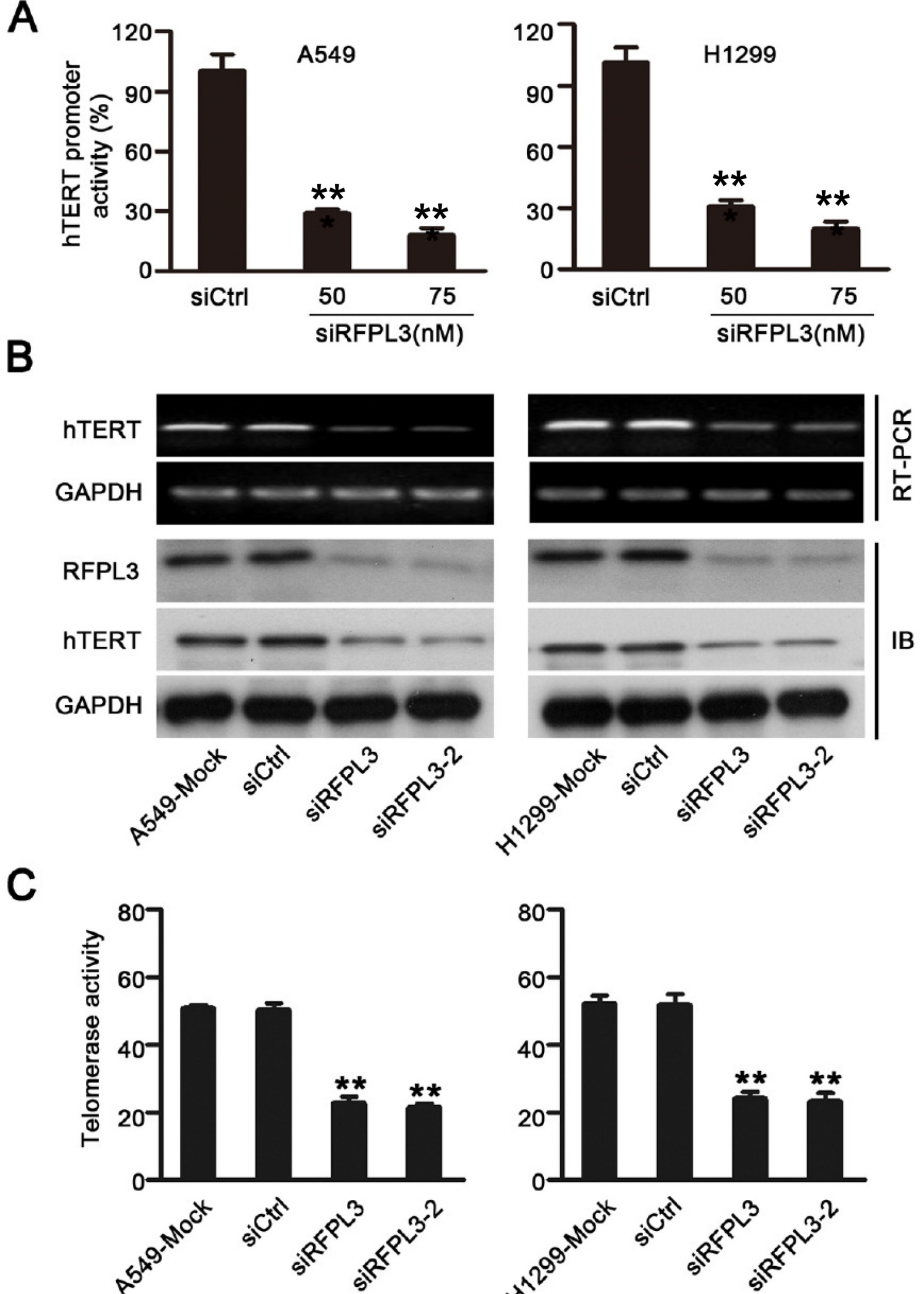
Knockdown of RFPL3 inhibits *hTERT* promoter activation and decreases hTERT expression and telomerase activity **(A)** The A549 and H1299 cells were co-transfected with the hTERT-luciferase plasmids and the indicated doses of RFPL3 siRNA or a non-specific control siRNA for 48 h. Luciferase activity was measured using a dual-luciferase assay. The inhibition of luciferase was calculated as a percentage relative to cells transfected with control siRNA. All of the measurements represent the means ± SE of three independent experiments (*P < 0.05). **(B)** The A549 and H1299 cells were transfected with RFPL3 siRNA or non-specific control siRNA for 48 h, and the expression of hTERT mRNA and protein was analyzed by RT-PCR and Western blot. **(C)** Telomerase activity was measured in A549 and H1299 cells by telomerase PCR enzyme-linked immunesorbent assay (**P < 0.01).

To further confirm the role of RFPL3 in the regulation of *hTERT* transcription and expression, we determined the effect of the inhibition of RFPL3 expression on *hTERT* promoter activity and expression. We transfected the RFPL3-specific siRNA into lung cancer cell lines (A549 and H1299) which express high levels of RFPL3. Our results showed that the blockade of RFPL3 by RFPL3-specific siRNA decreased the *hTERT* promoter-driven luciferase activity in A549 and H1299 cells (Fig. 3A). Consistently, the levels of hTERT mRNA and protein (Fig. 3B) and the telomerase activity (Fig. 3C) were also markedly suppressed in A549 and H1299 cells. These findings suggest that the knockdown of RFPL3 down-regulates *hTERT* promoter activity and hTERT expression in lung caner cells.

### Inhibition of tumor growth by RFPL3 shRNA *in vitro* and *in vivo*

**Figure 4:**
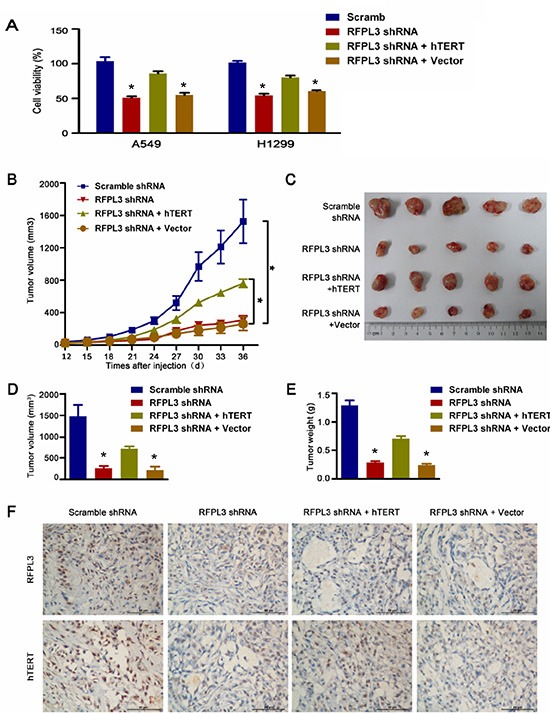
Knockdown of RFPL3 inhibits tumor growth by downregulating hTERT expression *in vitro* and *in vivo* **(A)** The stable A549 and H1299 cell lines were generated: (1) RFPL3 shRNA; (2) non-target control shRNA; (3) RFPL3 shRNA + hTERT; (4) RFPL3 shRNA + Control empty vector (EV). At 48 h after cells plated in 96-well plates, cell viability was measured using an MTT assay. The mean and SE obtained from three independent experiments are plotted. **(B)**Tumor growth curves: (*P < 0.05). **(C)** The harvested tumor grafts 36 days after inoculated subcutaneously into the flank of the nude mice. **(D)** Tumor volumes ± SE in nude mice. N = 5. **(E)** Mean tumor weights ± SE in nude mice. **(F)** Immunohistochemistry of RFPL3 and hTERT from tumor xenografts (400x magnification).

Since RFPL3 plays a positive role in the activation of hTERT expression and telomerase activity, we reasoned that RFPL3 may be a potential therapeutic target in lung adenocarcinoma treatment. So we sought to determine the effects of the RFPL3 knockdown on lung adenocarcinoma growth in *vitro* and in *vivo*. As shown in Fig. 4A, the knockdown of RFPL3 by stably expressing RFPL3 shRNA dramatically suppressed lung cancer cell proliferation in H1299 and A549 cells. To further investigate the involvement of hTERT expression in RFPL3 knockdown-mediated lung cancer cell proliferation suppression, we rescued hTERT expression using an hTERT-expressing lentivirus to infect the lung cancer cell lines stably expressing RFPL3 shRNA. We found that the RFPL3-specific shRNA-induced proliferation inhibition was partially rescued by the overexpression of hTERT (Fig. 4A).

To further assess whether RFPL3 is a potential target for lung cancer, we also evaluated tumorigenicity using a xenograft mouse model. Four stable cell lines: (1) RFPL3 shRNA; (2) non-target control shRNA; (3) RFPL3 shRNA + hTERT; (4) RFPL3 shRNA + Control empty vector (EV) were generated and implanted into nude mice, we found that tumor growth was significantly suppressed by the blockade of RFPL3 *in vivo* (Fig. 4B-E), and the RFPL3-specific shRNA-induced growth inhibition was partially rescued by the forced expression of hTERT (Fig. 4B-E). Moreover, as shown in Fig. 4F, the knockdown of RFPL3 by stably expressing RFPL3 shRNA did suppress the hTERT protein levels in xenografts tumor tissue. These results indicate that RFPL3 shRNA exerts its inhibitory effect on tumorigenicity partially through the downregulation of hTERT expression.

### Positive correlation between RFPL3 and hTERT expression in human lung adenocarcinoma tissues

**Figure 5:**
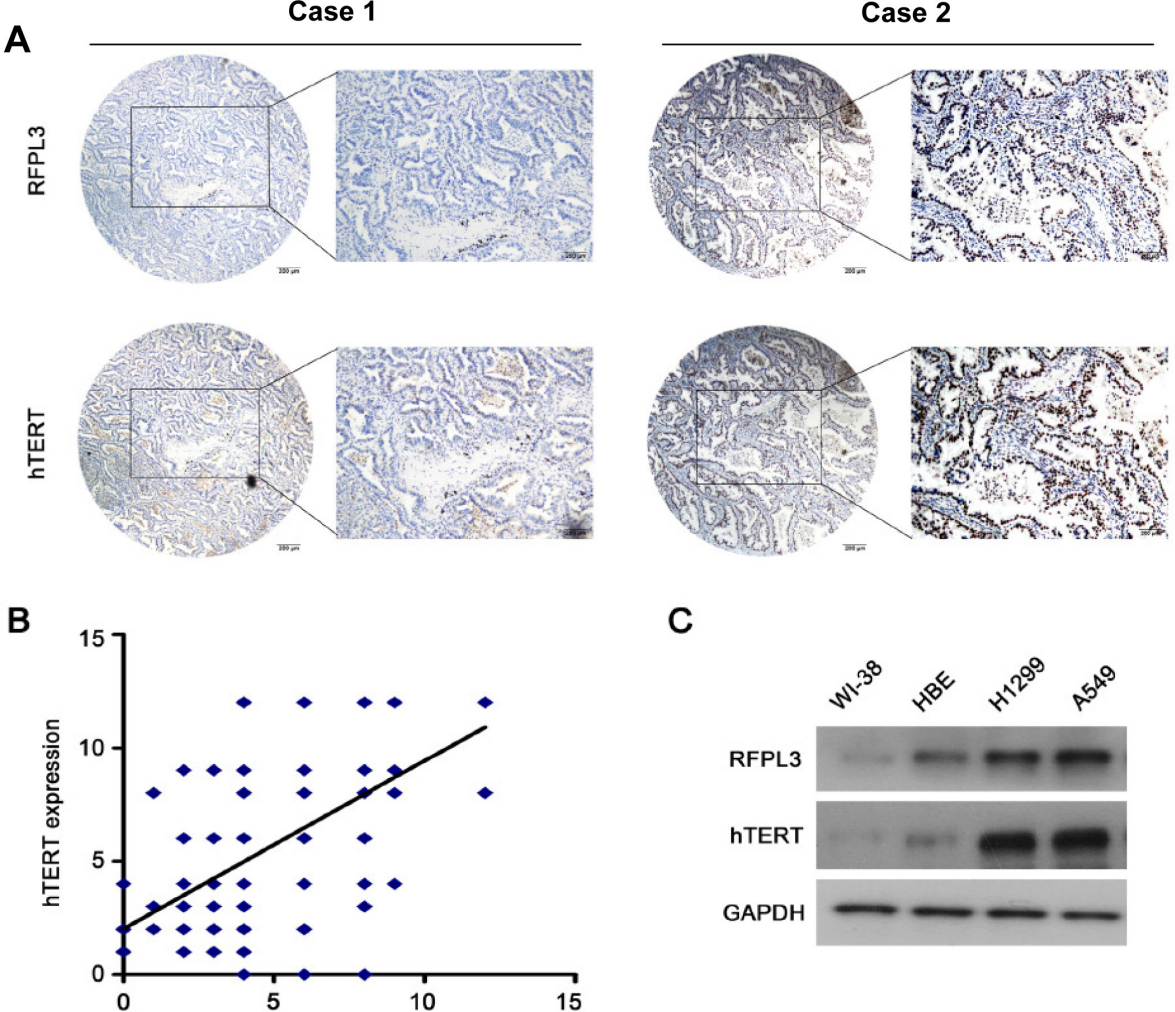
RFPL3 expression is positively correlated with hTERT expression in lung adenocarcinoma specimens and lung cancer cell lines **(A)** Representative immunohistochemical staining examples of low or high RFPL3 and hTERT expression in the serial sections from the same tumor tissues are shown. Scale bar, 200 μm. Case 1 and 2 means two different patients. **(B)** The tissue sections were quantitatively scored according to the percentage of positive cells and staining intensity as described in Materials and Methods. The percentage and intensity scores were multiplied to obtain a total score (range, 0–12). RFPL3 expression levels correlated positively with hTERT expression levels in lung adenocarcinoma samples (Pearson's correlation test, *r* = 0.639; *P* < 0.001). **(C)** The expression of RFPL3 and hTERT proteins in the total cell lysates of lung normal cells and cancer cells were analyzed by Western blot.

The expression of RFPL3 and hTERT proteins was further analyzed in 181 primary human lung adenocarcinoma tissues by IHC using tissue micro arrays (Fig. 5A). We scored the immunohistochemical staining of RFPL3 and hTERT in the human lung adenocarcinoma specimens by multiplying the intensity and the percentage value (the range of this calculation was therefore 0–12) and then analyzed the scores. A positive correlation was identified between expression of RFPL3 and hTERT by the Spearman correlation test (r = 0.639; *P* < 0.001) (Fig. 5B). Furthermore, we performed Western blot analyses using whole cell lysates from lung cancer and normal cell lines. As shown in Fig. 5C, RFPL3 expression correlated with hTERT expression. These results suggest that RFPL3 expression is positive correlated with hTERT expression.

### Association of RFPL3 overexpression with poor prognosis of lung adenocarcinoma patients

**Figure 6:**
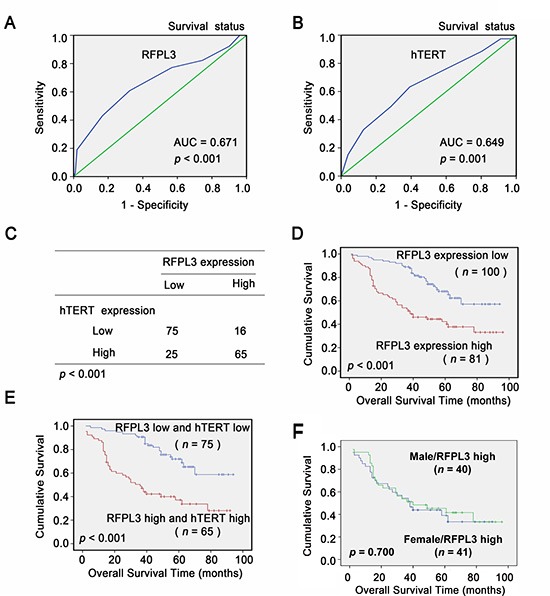
Overexpression of RFPL3 indicates a poor prognosis **A, B,** ROC curve analysis was used to determine the cut-off score for high expression of RFPL3 and hTERT protein in lung adenocarcinoma tissues. The sensitivity and specificity for OS were plotted: **(A)** RFPL3; *p* < 0.001 **(B)** hTERT; *p* = 0.001. **(C)** The protein level of RFPL3 correlates positively with the protein level of hTERT in lung adenocarcinoma tissues (*P* < 0.001, χ2 tests). **(D)** Kaplan–Meier analysis of overall survival with high RFPL3 expression ( *p* < 0.001, log-rank test). **(E)** Kaplan–Meier analysis of overall survival with high or low RFPL3 and hTERT expression ( *p* < 0.001, log-rank test). **(F)** Kaplan–Meier analysis of overall survival of male (n = 40) and female patients (n = 41) with high RFPL3 expression ( *p* = 0.700).

To develop a reasonable cut-off score of RFPL3 and hTERT for further survival analysis, we subjected each IHC score to ROC curve analysis with respect to patient outcome. As shown in (Fig. 6A and B), the RFPL3 and hTERT IHC cut-off scores for OS was 6.0. Thus, the expression of RFPL3 and hTERT in each sample was subsequently classified as either high level (score ≥ 6) or low level (score < 6). In the 181 human lung adenocarcinoma tissues, there was a positive correlation between the levels of RFPL3 protein and hTERT protein (*P* < 0.001, χ2 tests; Fig. 6C). The correlation between RFPL3 expression status and clinicopathologic features of lung adenocarcinoma was further evaluated, which was summarized in Table 1A. The results showed that RFPL3 upregulation was significantly associated with lymph node metastasis (*P* < 0.001, χ2 tests). No correlation was observed between RFPL3 upregulation and patient's gender (*P* = 0.829, χ2 tests), age (*P* = 0.673, χ2 tests), T classification (*P* = 0.617, χ2 tests). Kaplan–Meier analysis was used to study the survival curves in 181 patients with lung adenocarcinoma with survival data. Survival analyses showed that high expression of RFPL3 significantly correlated with shorter overall survival time in patients with lung adenocarcinomas (*P* < 0.001, log-rank test; Fig. 6D). Moreover, the lung adenocarcinoma patients with high RFPL3 and hTERT expression had a significantly shorter OS than those with low RFPL3 and hTERT expression (*P* < 0.001, log-rank test; Fig. 6E). By univariate analysis, RFPL3 upregulation (*P* < 0.001), T3 stage (*P* = 0.008) and presence of lymph node metastasis (*P* < 0.001) were significant inferior prognostic factors for OS in patients with lung adenocarcinoma (Table 1B). Multivariate analysis further indicated that RFPL3 upregulation (*P* = 0.001) and presence of lymph node metastasis (*P* = 0.002) were 2 independent prognostic predictors for OS in patients with lung adenocarcinoma enrolled in this study (Table 1B). These results suggest that RFPL3 upregulation is an independent prognostic factor for OS of lung adenocarcinomas patients. Survival analyses also demonstrated that there was no significant difference of the overall survival time between male and female patients with high expression of RFPL3 protein (Fig. 6F).

**Table 1A:** Association of RFPL3 expression with patient's clinicopathological features in lung ADC

	Total (*n* = 181)	RFPL3 low expression (*n* = 100)	RFPL3 high expression (*n* = 81)	*p*
Gender				
Male	91	50(54.9%)	41(45.1%)	0.829
Female	90	49(54.4%)	41(45.6%)	
Age, y				
<60	97	55(56.7%)	42(43.3%)	0.673
≥60	84	45(53.6%)	39(46.4%)	
pT factor				
T1+ T2	161	90(55.9%)	71(44.1%)	0.617
T3	20	10(50%)	10(50%)	
pN factor				
N0	126	82(65.1%)	44(34.9%)	<0.001^a^
N1+N2	55	18(32.7%)	37(67.3%)	

Abbreviations: ADC, adenocarcinoma; ^a^*P* < 0.05

**Table 1B:** Cox proportional hazards model analysis of prognostic factors in patients with lung ADC

	HR	95% CI	Unfavorable/Favorable	*p*
Univariate analysis				
RFPL3	2.678	1.700–4.216	High/low	<0.001^a^
Gender	0.823	0.529–1.280	Male/female	0.387
Age,y	1.232	0.791–1.917	≥60/<60	0.356
pT factor	2.152	1.225–3.781	T3/T1+T2	0.008a
pN factor	2.891	1.845–4.530	N1+N2/N0	<0.001^a^
Multivariate analysis				
RFPL3	2.181	1.364–3.487	High/low	0.001^a^
pT factor	1.535	0.860–2.741	T3/T1+T2	0.147
pN factor	2.157	1.337–3.481	N1+N2/N0	0.002^a^

Abbreviations: ADC, adenocarcinoma; HR, hazard ratio; CI, confidence interval; ^a^*P* < 0.05

## DISCUSSION

Many high-throughput methods were used to develop molecular-targeting anticancer drugs that are expected to be highly specific to cancer cells, with minimal risk of adverse reactions on normal cells [29, 30]. One of the methods is systematic analysis of expression levels of thousands of genes between malignant and normal cells by a cDNA microarray technology. Here, we established an effective system to develop potential molecular targets for diagnosis and/or treatment of cancers by identifying proteins that bind to and activate promoter of carcinogenic genes specifically in cancers. Firstly, we isolated large complex of proteins that bind to carcinogenic genes promoter from cancer and normal cells by the streptavidin-agarose pulldown and identified the candidate proteins by proteomics. Then we investigated the role of candidate proteins in regulating promoter activation of carcinogenic genes. Using this approach, we discovered novel expression regulating mechanisms of carcinogenic genes *COX-2* and *hTERT* [22–24, 28]. Previously AP-2β was identified as a tumor-specific *hTERT* promoter binding protein in NSCLC [28]. And AP-2β was verified as an independent predictor of poor overall survival in patients with stage I NSCLC undergoing curative resection [31]. AP-2β was also a helpful diagnostic marker for discrimination between embryonal rhabdomyosarcoma and alveolar rhabdomyosarcoma upon diagnosing a child with rhabdomyosarcoma and a predictor prognosis marker for unfavorable overall survival [32–34]. In this study, we detected and identified, for the first time, RFPL3 as a novel hTERT promoter binding proteins by the approach. Furthermore, our *in vitro* and *in vivo* results revealed that RFPL3 knockdown inhibited lung cancer cell growth and the genetic–clinicopathologic correlation analysis found that upregulation of RFPL3 was significantly associated with shorter OS of patients with lung adenocarcinomas. Therefore, our results once again suggest that the approach combined streptavidin-agarose pulldown assay and proteomics is a useful approach to screen potential molecular targets for the diagnosis and/or treatment of cancers.

hTERT plays a pivotal role in cells immortalization and malignant transformation [8]. The hTERT is overexpressed in 80–95% of cancers and so it is known as a hallmarker of tumor. In NSCLC, hTERT protein was mainly localized in the nuclei of cancer cells and its overexpression was a poor prognostic marker for OS and disease-free survival [35, 36]. However, little is known about the underlying molecular mechanisms of the reactivation of hTERT during tumorigenesis for NSCLC. Studies have indicated that *hTERT* gene amplification is one of the mechanisms for hTERT overexpression in NSCLC [37]. But *hTERT* gene amplification can not completely account for the cancer-specific overexpression of hTERT in NSCLC. Here, for the first time, we showed that the increased abundance of RFPL3 protein is another potential molecular mechanism for the up-regulated hTERT expression in some lung adenocarcinomas by supporting both clinical and mechanistic evidence. However, the upregulation of RFPL3 protein also can not completely account for the cancer-specific overexpression of hTERT in NSCLC. Because we observed that there were 25 tumors that contained high levels of hTERT and low levels of RFPL3 in our study (Fig.6C), indicating that other factors, in addition to the RFPL3, may be involved in the upregulation of hTERT in lung adenocarcinomas. For example, AP-2β activates the expression of the hTERT in lung cancer, as we reported previously [28]. In consideration of that previous studies on the regulation of hTERT expression have revealed that regulation of the *hTERT* gene was mainly at the transcriptional level and was very diversity and complexity [14], appearance of this kind of deviation is not difficult to understand.

By analyzing the function structural domain of RFPL3, we found that it is lack of DNA-binding domains which is frequently needed for binding to target gene promoter. Each of the domains of RFPL3 has been suggested to mediate protein–protein interactions [38]. It has been reported that the RFP regulated *TBP-2* gene expression by recruiting the Histone deacetylases 1 and the trimeric transcription factor NF-Y to the *TBP-2* promoter in colon cancer cells [39]. Given the highest similarity in the structure of RFPL3 and RFP [38], we speculated that RFPL3 may also execute its co-activation effect on *hTERT* expression by recruiting the transcription factors to bind to the *hTERT* promoter. Further detailed analyses are necessary to determine the partner transcription factors of RFPL3 on *hTERT* expression activation in lung adenocarcinomas. Thus, for the first time, our report describes a novel molecular mechanism by which RFPL3 promotes *hTERT* transcription and expression in lung adenocarcinomas.

*RFPL3* gene locates at human 22q12.3, belongs to the RFPL proteins family which plays an important regulatory role in embryonic development [25, 26]. RFPL1 inhibited HeLa cells proliferation through delaying cells entry into mitosis [27]. However, the molecular expression and the function of other RFPL proteins family members including RFPL3 in cancers are still unclear. Our results showed that the overexpression of RFPL3 protein was in 45% of primary lung adenocarcinomas. We demonstrate here that high level of RFPL3 protein expression in tumor cells is associated with lymph node metastasis and poor outcome in patients with lung adenocarcinoma. To our knowledge, this is the first study to report a role of RFPL3 in lung adenocarcinomas. However, the exact molecular mechanism responsible for RFPL3 overexpression in lung adenocarcinoma cells is still unknown. The 22q11-q13 region, which corresponds to the chromosomal location of the RFPL3 gene, was enriched in copy-number variations [25]. Meanwhile, it has been reported that RFPL3 was transactivated by the transcription factor Pax6 [25], its mRNA was highly expressed in primary NSCLC tissue compared to their matched adjacent tissues [40]. We speculated that the amplification and transcription activation by the overexpression of transcription factor Pax6 of *RFPL3* gene maybe responsible for *RFPL3* overexpression. Further detailed analyses are necessary to determine the underlying molecular mechanism of *RFPL3* overexpression.

In summary, our findings show that RFPL3 is a novel *hTERT* promoter regulating protein and plays an important role in the hTERT overexpression and tumor growth in lung adenocarcinomas. Our results suggest that RFPL3 might be a potential therapeutic target in lung adenocarcinomas.

## MATERIALS AND METHODS

### Cell lines, stable cell lines and plasmids

The human adenocarcinoma cell lines (H1299, A549) were obtained from the American Type Culture Collection and cultured in RPMI-1640 medium (Invitrogen, Carlsbad, CA) supplemented with 10% fetal bovine serum. Two human normal lung cell lines (fibroblast WI-38 and epithelial lung cell HBE) were cultured in Dulbecco's Modified Eagle Medium (Invitrogen, Carlsbad, CA) supplemented with 10% fetal bovine serum. All cells were maintained in a humidified atmosphere and 5% CO_2_ at 37°C.

The lentivirus particles for RFPL3 short-hairpin RNA (shRNA) or the scrambled non-target control shRNA were purchased from Santa Cruz Biotechnology (Santa Cruz, CA). The lentivirus particles and plasmid for RFPL3 and hTERT overexpression were designed and synthesized by Cyagen (Cyagen Biosciences Inc., United States). H1299 and A549 were used to generate stable cell lines in this study. To knockdown RFPL3 expression, the lung cancer cell lines stably expressing RFPL3 shRNA were established. To rescue hTERT expression, an hTERT-expressing lentivirus was used to co-infect the lung cancer cell lines stably expressing RFPL3 shRNA. A fragment of the human hTE*RT* promoter (−400 to +60) were cloned to pGL3 basic vector (Promega Corp., Madison, WI) to generate the *hTERT* promoter luciferase plasmid pGL3-hTERT-400.

### DNA-protein binding by streptavidin-agarose pulldown assay

Transactivators binding to an *hTERT* core promoter probes were determined by a streptavidin-agarose pulldown assay. Briefly, a biotin-labeled double-stranded DNA probe corresponding to nucleotides −378 to +60 of the *hTERT* promoter sequence was synthesized by Sigma (Sigma-Aldrich, St. Louis, MO). One mg of cells nuclear protein extract, 10 ug of DNA probe and 100 ul of streptavidin-agarose beads (Sigma-Aldrich) were mixed and incubated at room temperature for 2 h with a rotating shaker and then pelleted by centrifugation to pull down the DNA-protein complex. After washing with cold phosphate-buffered saline (PBS), proteins in the complex were analyzed by further assay.

### Identification of *hTERT* promoter-binding proteins

The bound proteins complex were separated by 4–15% SDS-PAGE and visualized by silver staining. The protein bands of interests were cut out and digested with sequencing-grade trypsin solution and identified using mass spectrum. The identities of the proteins of interest were verified via available databases and software.

### Chromatin immunoprecipitation assay (ChIP)

The ChIP assay was performed using the ChIP IT Express kit (Active Motif, Rixensart, Belgium) as described previously [41]. Briefly, the cells were fixed with 1% formaldehyde and sonicated and then subjected to immunoprecipitation with anti-RFPL3 antibodies or non-immune rabbit IgG (Abcam). The specific *hTERT* promoter primers were as follows: 5′-TGGCCCCTCCCTCGGGTTAC-3′ and 5′-CCAGGGCTTCCCACGTGCGC-3′. The resulting product of 438-bp for *hTERT* in length was separated by 2% agarose gel electrophoresis and visualized by ethidium bromide staining.

### Transient transfection

The cells were transfected with a RFPL3 express vector or a control vector using Lipofectamine 2000 (Invitrogen, Carlsbad, CA). To inhibit RFPL3 expression, A549 cells and H1299 cells were transfected with siRNA oligonucleotides. The oligos corresponding to RFPL3 were termed siRFPL3 (5′-GUG GGA ACA AGC ACA GAA UTT-3′) and siRFPL3-2 (5′-CGC UGA CUU UCC UCU UAG UTT-3′); the sequence of the nonspecific siRNA was 5′-UUC UCC GAA CGU GUC ACG UTT-3′. These siRNA oligonucleotides were purchased from Shanghai GenePharma Co. (Shanghai China). Forty-eight hours after transfection, RNA and protein were isolated, and RT-PCR, telomerase activity and western blot analyses were carried out as described below.

### Dual-luciferase assay

To study the effects of RFPL3 on *hTERT* promoter activity, the dual luciferase reporter assay (Promega) was performed using the Dual-Luciferase Assay Kit (Promega Corp., Madison, WI).

### Telomerase activity assays

Telomerase activity was analyzed by a telomerase PCR enzyme-linked immunosorbent assay kit (Roche Applied Science).

### Cell viability assay

Cell viability was determined using an MTT assay kit (Roche Diagnosis, Indianapolis, IN) according to the manufacturer's protocol. Briefly, stable cell lines were plated in 96-well plates (2, 000 cells/well) and cell viability was determined after 48 h.

### Western blot analysis

Western blot analyses of the cell lysates were performed with antibodies against RFPL3 (Abcam), hTERT (Epitomics). Immunoreactive protein bands were detected using an enhanced chemiluminescence kit (Amersham Pharmacia Biotech, Piscataway, NJ) according to the manufacturer's instructions.

### RT-PCR

Isolation of the total cellular RNA was performed using TRIzol reagent (Life Technologies, Glasgow, UK) according to the manufacturer's instructions. cDNA was synthesized and used for amplification of *hTERT* gene. The primers were as follows: 5′-GTCGAGCTGCTCAGGTCTT-3′ and 5′-AGTGCTGTCTGATTCCAATGCTT-3′. The PCR products was separated by agarose gel electrophoresis and visualized by ethidium bromide staining.

### *In vivo* tumorigenicity assays and xenograft tumor tissues processing

Animal experiments were carried out in accordance with the National Institute of Health Guide for the Care and Use of Laboratory Animals, with the approval of the Animal Research Committee of Sun Yat-sen University Cancer Center, Guangzhou. Four stable A549 cell lines (2 × 10^6^) were respectively inoculated subcutaneously into the flank of the nude mice (5 mice per group): (1) RFPL3 shRNA; (2) non-target control shRNA; (3) RFPL3 shRNA + hTERT; (4) RFPL3 shRNA + Control empty vector (EV). Once palpable tumors were observed, tumor volume measurements were taken every 3 days using calipers. The tumor volume was calculated as V = width^2^ × length × 0.5 using digital calipers. The mice were sacrificed, and the tumor weight and tumor volume was recorded. The xenograft tumor tissues were fixed in formalin and embedded in paraffin for RFPL3 and hTERT protein expression analysis. The immunohistochemical staining was performed as described below.

### Human lung adenocarcinoma tissue microarray

The human lung adenocarcinoma tissue microarray used for immunostaining analysis of RFPL3 and hTERT protein expression was purchased from Shanghai Outdo Biotech (Shanghai, China) and contains 181 formalin-fixed, paraffin-embedded lung adenocarcinomas and their corresponding adjacent non-malignant lung tissues. No patients recruited in this study have received any preoperative treatment. These tissue samples had been obtained with prior written consent from patients.

### Immunohistochemistry (IHC) staining

The IHC staining procedure was performed as described previously [42]. In brief, the slides were deparaffinized, rehydrated and blocked by 10% normal goat serum at room temperature for 30 minutes. The slides were then incubated with a primary antibody, either anti-RFPL3 (1:50 dilution), or anti-hTERT antibody (1:50 dilution), in a humidified container at 4°C. RFPL3 and hTERT demonstrated predominantly nuclear staining in the cancer cells. For each marker, two specialist lung pathologists independently calculated the percentage staining and the predominant intensity on a predetermined scale as follows. Nuclear staining intensity was graded as follows: absent staining as 0, weak as 1, moderate as 2, and strong as 3. The percentage of stained cells was graded as follows: 0 (no positive cells), 1 (<25% positive cells), 2 (25%–50% positive cells), 3 (50%–75% positive cells), and 4 (>75% positive cells). The score for each tissue was calculated by multiplying the intensity and the percentage value (the range of this calculation was therefore 0–12). The receiver operating characteristic (ROC) curve analysis was employed to determine cutoff score for tumor “high expression” by using the 0, 1-criterion. Tumors designated as “low expression” for RFPL3 and hTERT were those with scores below the cutoff value, while “high expression” tumors were those with scores equal to or above the value.

### Statistical analysis

Student's t-tests were used to compare two independent groups of data. Chi-square tests were applied to analyze the correlation between RFPL3 expression and clinicopathologic features of lung adenocarcinomas patients. Survival curves were constructed using the Kaplan-Meier method and were compared using the log-rank test. Multivariate Cox proportional hazards analyses used “backwards” modeling to generate models predictive of outcome. Spearman correlation was used to explore the relationship between the abundance of RFPL3 and hTERT. Statistical analyses were performed using the SPSS 16.0 software. The results were reported as the mean ± SE. Values of *P* < 0.05 were considered to be statistically significant.

## References

[1] Siegel R, Naishadham D, Jemal A (2013). Cancer statistics, 2013. CA: a cancer journal for clinicians.

[2] Maemondo M, Inoue A, Kobayashi K, Sugawara S, Oizumi S, Isobe H, Gemma A, Harada M, Yoshizawa H, Kinoshita I, Fujita Y, Okinaga S, Hirano H, Yoshimori K, Harada T, Ogura T (2010). Gefitinib or chemotherapy for non-small-cell lung cancer with mutated EGFR. The New England journal of medicine.

[3] Shaw AT, Yeap BY, Mino-Kenudson M, Digumarthy SR, Costa DB, Heist RS, Solomon B, Stubbs H, Admane S, McDermott U, Settleman J, Kobayashi S, Mark EJ, Rodig SJ, Chirieac LR, Kwak EL (2009). Clinical features and outcome of patients with non-small-cell lung cancer who harbor EML4-ALK. Journal of clinical oncology: official journal of the American Society of Clinical Oncology.

[4] Nebhan C, Pao W (2013). Further advances in genetically informed lung cancer medicine. Journal of thoracic oncology: official publication of the International Association for the Study of Lung Cancer.

[5] Pao W, Girard N (2011). New driver mutations in non-small-cell lung cancer. The lancet oncology.

[6] Remon J, Moran T, Reguart N, Majem M, Carcereny E, Lianes P (2014). Beyond EGFR TKI in EGFR-mutant Non-Small Cell Lung Cancer patients: Main challenges still to be overcome. Cancer treatment reviews.

[7] Shaw AT, Kim DW, Nakagawa K, Seto T, Crino L, Ahn MJ, De Pas T, Besse B, Solomon BJ, Blackhall F, Wu YL, Thomas M, O'Byrne KJ, Moro-Sibilot D, Camidge DR, Mok T (2013). Crizotinib versus chemotherapy in advanced ALK-positive lung cancer. The New England journal of medicine.

[8] Ruden M, Puri N (2013). Novel anticancer therapeutics targeting telomerase. Cancer treatment reviews.

[9] Shay JW, Keith WN (2008). Targeting telomerase for cancer therapeutics. British journal of cancer.

[10] Agrawal A, Dang S, Gabrani R (2012). Recent patents on anti-telomerase cancer therapy. Recent patents on anti-cancer drug discovery.

[11] Tian X, Chen B, Liu X (2010). Telomere and telomerase as targets for cancer therapy. Applied biochemistry and biotechnology.

[12] Blackburn EH (2005). Telomerase and Cancer: Kirk A. Landon–AACR prize for basic cancer research lecture. Molecular cancer research: MCR.

[13] Shay JW, Wright WE (2006). Telomerase therapeutics for cancer: challenges and new directions. Nature reviews Drug discovery.

[14] Kyo S, Takakura M, Fujiwara T, Inoue M (2008). Understanding and exploiting hTERT promoter regulation for diagnosis and treatment of human cancers. Cancer science.

[15] Yu K, Zheng B, Han M, Wen JK (2011). ATRA activates and PDGF-BB represses the SM22alpha promoter through KLF4 binding to, or dissociating from, its cis-DNA elements. Cardiovascular research.

[16] Lu B, Lee J, Nie X, Li M, Morozov YI, Venkatesh S, Bogenhagen DF, Temiakov D, Suzuki CK (2013). Phosphorylation of human TFAM in mitochondria impairs DNA binding and promotes degradation by the AAA+ Lon protease. Molecular cell.

[17] Ramakrishnan P, Clark PM, Mason DE, Peters EC, Hsieh-Wilson LC, Baltimore D (2013). Activation of the transcriptional function of the NF-kappaB protein c-Rel by O-GlcNAc glycosylation. Science signaling.

[18] Badding MA, Lapek JD, Friedman AE, Dean DA (2013). Proteomic and functional analyses of protein-DNA complexes during gene transfer. Molecular therapy: the journal of the American Society of Gene Therapy.

[19] Powers NR, Eicher JD, Butter F, Kong Y, Miller LL, Ring SM, Mann M, Gruen JR (2013). Alleles of a polymorphic ETV6 binding site in DCDC2 confer risk of reading and language impairment. American journal of human genetics.

[20] Bu Y, Gao L, Gelman IH (2011). Role for transcription factor TFII-I in the suppression of SSeCKS/Gravin/Akap12 transcription by Src. International journal of cancer Journal international du cancer.

[21] Picard C, Pellicelli M, Taheri M, Lavoie JF, Doucet R, Wang D, Bernard L, Bouhanik S, Lavigne P, Moreau A (2013). Nuclear accumulation of prohibitin 1 in osteoarthritic chondrocytes down-regulates PITX1 expression. Arthritis and rheumatism.

[22] Deng WG, Wu KK (2003). Regulation of inducible nitric oxide synthase expression by p300 and p50 acetylation. J Immunol.

[23] Deng WG, Tang ST, Tseng HP, Wu KK (2006). Melatonin suppresses macrophage cyclooxygenase-2 and inducible nitric oxide synthase expression by inhibiting p52 acetylation and binding. Blood.

[24] Deng WG, Zhu Y, Wu KK (2004). Role of p300 and PCAF in regulating cyclooxygenase-2 promoter activation by inflammatory mediators. Blood.

[25] Bonnefont J, Nikolaev SI, Perrier AL, Guo S, Cartier L, Sorce S, Laforge T, Aubry L, Khaitovich P, Peschanski M, Antonarakis SE, Krause KH (2008). Evolutionary forces shape the human RFPL1,2,3 genes toward a role in neocortex development. American journal of human genetics.

[26] Assou S, Boumela I, Haouzi D, Monzo C, Dechaud H, Kadoch IJ, Hamamah S (2012). Transcriptome analysis during human trophectoderm specification suggests new roles of metabolic and epigenetic genes. PloS one.

[27] Bonnefont J, Laforge T, Plastre O, Beck B, Sorce S, Dehay C, Krause KH (2011). Primate-specific RFPL1 gene controls cell-cycle progression through cyclin B1/Cdc2 degradation. Cell death and differentiation.

[28] Deng WG, Jayachandran G, Wu G, Xu K, Roth JA, Ji L (2007). Tumor-specific activation of human telomerase reverses transcriptase promoter activity by activating enhancer-binding protein-2beta in human lung cancer cells. The Journal of biological chemistry.

[29] Aragaki M, Takahashi K, Akiyama H, Tsuchiya E, Kondo S, Nakamura Y, Daigo Y (2011). Characterization of a cleavage stimulation factor, 3′ pre-RNA, subunit 2, 64 kDa (CSTF2) as a therapeutic target for lung cancer. Clinical cancer research: an official journal of the American Association for Cancer Research.

[30] Daigo Y, Nakamura Y (2008). From cancer genomics to thoracic oncology: discovery of new biomarkers and therapeutic targets for lung and esophageal carcinoma. General thoracic and cardiovascular surgery.

[31] Kim MP, Chen Y, Bekele BN, Lopez A, Khanna A, Chen JQ, Spitz MR, Behrens C, Solis L, Wismach M, Ji L, Wistuba II, Roth JA, Katz RL (2011). Activating enhancer-binding protein-2beta nucleolar localization predicts poor survival after stage I non-small cell lung cancer resection. The Annals of thoracic surgery.

[32] Grass B, Wachtel M, Behnke S, Leuschner I, Niggli FK, Schafer BW (2009). Immunohistochemical detection of EGFR, fibrillin-2, P-cadherin and AP2beta as biomarkers for rhabdomyosarcoma diagnostics. Histopathology.

[33] Rudzinski ER, Anderson JR, Lyden ER, Bridge JA, Barr FG, Gastier-Foster JM, Bachmeyer K, Skapek SX, Hawkins DS, Teot LA, Parham DM (2014). Myogenin, AP2beta, NOS-1, and HMGA2 Are Surrogate Markers of Fusion Status in Rhabdomyosarcoma: A Report From the Soft Tissue Sarcoma Committee of the Children's Oncology Group. The American journal of surgical pathology.

[34] Wachtel M, Rakic J, Okoniewski M, Bode P, Niggli F, Schafer BW (2014). FGFR4 signaling couples to Bim and not Bmf to discriminate subsets of alveolar rhabdomyosarcoma cells. International journal of cancer Journal international du cancer.

[35] Wang L, Soria JC, Kemp BL, Liu DD, Mao L, Khuri FR (2002). hTERT expression is a prognostic factor of survival in patients with stage I non-small cell lung cancer. Clinical cancer research: an official journal of the American Association for Cancer Research.

[36] Lantuejoul S, Soria JC, Moro-Sibilot D, Morat L, Veyrenc S, Lorimier P, Brichon PY, Sabatier L, Brambilla C, Brambilla E (2004). Differential expression of telomerase reverse transcriptase (hTERT) in lung tumours. British journal of cancer.

[37] Zhu CQ, Cutz JC, Liu N, Lau D, Shepherd FA, Squire JA, Tsao MS (2006). Amplification of telomerase (hTERT) gene is a poor prognostic marker in non-small-cell lung cancer. British journal of cancer.

[38] Zhang G, Wang G, Wang S, Li Q, Ouyang G, Peng X (2004). Applying proteomic methodologies to analyze the effect of hexamethylene bisacetamide (HMBA) on proliferation and differentiation of human gastric carcinoma BGC-823 cells. The international journal of biochemistry & cell biology.

[39] Kato T, Shimono Y, Hasegawa M, Jijiwa M, Enomoto A, Asai N, Murakumo Y, Takahashi M (2009). Characterization of the HDAC1 complex that regulates the sensitivity of cancer cells to oxidative stress. Cancer research.

[40] Zhao X, Yue W, Zhang L, Ma L, Jia W, Qian Z, Zhang C, Wang Y (2014). Downregulation of PAX6 by shRNA inhibits proliferation and cell cycle progression of human non-small cell lung cancer cell lines. PloS one.

[41] Xiao X, Shi D, Liu L, Wang J, Xie X, Kang T, Deng W (2011). Quercetin suppresses cyclooxygenase-2 expression and angiogenesis through inactivation of P300 signaling. PloS one.

[42] Shi D, Xie F, Zhang Y, Tian Y, Chen W, Fu L, Wang J, Guo W, Kang T, Huang W, Deng W (2014). TFAP2A regulates nasopharyngeal carcinoma growth and survival by targeting HIF-1alpha signaling pathway. Cancer Prev Res (Phila).

